# Supplementary material for: Predictors and prevalence of labor pain management practice at West Guji zone, Southern Ethiopia, 2024
Source: PLOS Glob Public Health. 2026 Jan 5;6(1):e0005727. doi: 10.1371/journal.pgph.0005727 (PMC12768246; doi:10.1371/journal.pgph.0005727)
Supplement: Attachment — Submitted filename: Plos GPH_REVIEWERS_TG.docx [file pgph.0005727.s002.docx]

**Manuscript Number: PGPH-D-25-02053**

**PREDICTORS AND PREVALENCE OF LABOR PAIN MANGEMENT PRACTICE AT WEST GUJI ZONE, SOUTHERN ETHIOPIA, 2025.**

Thank you for letting me review this manuscript. It is a relevant topic, but requires a revision before it can be considered for publication.

- **The Clarity and Language**: The manuscript requires a comprehensive review to address grammatical errors, punctuation, and overall clarity, ensuring the scientific message is conveyed effectively. E.g., on the title, short title, and keywords, the word ‘Management’ was misspelled.
- **Abstract**:
  - **Introduction:** Try to convey your message with scientifically sound statements (rather than using ‘Strategies for managing labor pain include both non-drug methods and medical interventions.’ Better to replace with non-pharmacological and pharmacological…. ). Be focused on the major title (I didn’t see the importance of the statement mentioned… ‘Over one-third of maternal fatalities resulting from pregnancy-related issues are linked to complications that occur during delivery or shortly after, often caused by hemorrhage, obstructed labor, or sepsis’ for this study/no association with your study.
  - **Results**: make it clear whether the magnitude (43.3%) stated is for pharmacological or non-pharmacological labor pain management or both. Again, it is not rate, rather it is magnitude/prevalence.
  - **Conclusion**: at least indicate one recommendation based on your findings.
- **Introduction**: make it short and focused. The rationale for conducting this study is not sufficiently strong. The authors should more clearly state what unique gap in the literature this research addresses, considering the existing studies from other parts of Ethiopia
- **Methods**
  - **Sampling methods**: your sample size is not clear, 415 or 280? Please revise this section accordingly. The text states, "415 study participants were chosen using a simple random selection procedure," but the numbers provided (185 from hospitals + 230 from health centers = 415) do not match the adjusted sample size of 280. This discrepancy must be resolved
  - **Data collection Procedure:** You mentioned that the data were collected by health professionals working in the same institution, so how did you treat the bias associated with this?
  - Provide more details on the data collection tool and validation.
- **Results**:
  - When you use abbreviations and “Other,” indicate the full list as a footnote at the end of each table.
  - **Prevalence of labor pain management Practice:** please make it clear which type of labor pain management was practiced in the statement…As a result, 116 (43.3%) of the total respondents provided labor pain management to customers for laboring mothers, whereas 152 (56.7%) did not….
  - **Figure 2**: I didn’t see the importance of Figure 2, since you already stated the finding in a statement. I recommend removing it.
  - The Adjusted Odds Ratio (AOR) for "knowledge of obstetric analgesia" is reported as 9.4; 95% CI: 1.8, 9.9. The upper confidence limit (9.9) is very close to the point estimate (9.4), which is highly unusual and suggests a possible calculation or reporting error. Furthermore, a 95% CI that spans from 1.8 to 9.9 is extremely wide, indicating substantial imprecision in this estimate. This must be verified and corrected also in the abstract section.
  - I suggest presenting knowledge and attitude data in a organized **table** to show the specific variables examined in these sections..
- **Discussion**:
  - The discussion does not adequately interpret the key findings. For example, the strong association with knowledge is logical, but the negative association with holding a diploma (AOR 0.3) is a crucial finding that deserves deeper exploration. Why would less-educated providers be less likely to manage pain? Is it a proxy for training, confidence, or institutional role?
- **Conclusion**: needs revision
  - Conclusion is not only a place for the indication of your results, but also needs recommendations. Please describe some recommendations based on your study findings.
- **Data Availability Statement:** The data availability statement is missing from the manuscript text, though it is a requirement per the journal's submission system.
